# Cyto-adherence of *Mycoplasma mycoides* subsp. *mycoides* to bovine lung epithelial cells

**DOI:** 10.1186/s12917-015-0347-3

**Published:** 2015-02-07

**Authors:** Racheal Aye, Martin Kiogora Mwirigi, Joachim Frey, Paola Pilo, Joerg Jores, Jan Naessens

**Affiliations:** International Livestock Research Institute, P. O. Box 30709-00100, Nairobi, Kenya; Institute of Veterinary Bacteriology, University of Bern, Bern, Switzerland

**Keywords:** *Mycoplasma mycoides* subsp. *mycoides*, Contagious bovine pleuropneumonia, Cyto-adherence, Epithelial cells

## Abstract

**Background:**

*Mycoplasma mycoides* subsp. *mycoides* (*Mmm*) is the causative agent of contagious bovine pleuropneumonia (CBPP), a respiratory disease of cattle, whereas the closely related *Mycoplasma mycoides* subsp. *capri* (*Mmc*) is a goat pathogen. Cyto-adherence is a crucial step in host colonization by mycoplasmas and subsequent pathogenesis. The aim of this study was to investigate the interactions between *Mmm* and mammalian host cells by establishing a cyto-adherence flow cytometric assay and comparing tissue and species specificity of *Mmm* and *Mmc* strains.

**Results:**

There were little significant differences in the adherence patterns of eight different *Mmm* strains to adult bovine lung epithelial cells. However, there was statistically significant variation in binding to different host cells types. Highest binding was observed with lung epithelial cells, intermediate binding with endothelial cells and very low binding with fibroblasts, suggesting the presence of effective adherence of *Mmm* on cells lining the airways of the lung, which is the target organ for this pathogen, possibly by high expression of a specific receptor. However, binding to bovine fetal lung epithelial cells was comparably low; suggesting that the lack of severe pulmonary disease seen in many infected young calves can be explained by reduced expression of a specific receptor.

**Conclusions:**

*Mmm* bound with high efficiency to adult bovine lung cells and less efficiently to calves or goat lung cells. The data show that cyto-adherence of *Mmm* is species- and tissue- specific confirming its role in colonization of the target host and subsequent infection and development of CBPP.

**Electronic supplementary material:**

The online version of this article (doi:10.1186/s12917-015-0347-3) contains supplementary material, which is available to authorized users.

## Background

*Mycoplasma* species (class *Mollicutes*) are widespread parasites of man, animals, plants and insects that are considered typical surface parasites [1]. *Mycoplasma* species have a small genome and are the smallest self-replicating organisms [2]. Owing to their limited biosynthetic capabilities, most *Mycoplasma* species exhibit strict host and tissue specificities [2]. Due to lack of a cell wall, mycoplasmal adhesins must be part of their cell membrane allowing for direct contact between the mycoplasmal cell membrane and specific receptors on the host cell membrane [3]. This close interaction probably creates a micro-environment which allows for the uptake of important nutrients and accumulation of mycoplasmal metabolic end products causing damage to host cell membranes [1]. Adhesion mechanisms of *M. pneumoniae* [4] and *M. genitalium* [5] are the best studied among *Mycoplasma* species. In *M. genitalium* defective mutants of the adhesions P1 and P30 have been shown to be avirulent, hence considering them as major virulence factors, [6]. For *M. conjunctivae*, adhesion was shown to depend on the RGD motif (Arg-Gly-Asp) of Lipoprotein T (LppT), a protein belonging to the α-integrin binding lectin family that includes fibronectin, vibronectin, fibrillin and von Willebrand factor [7]. No adhesion molecules of *Mycoplasma mycoides* subsp. *mycoides* (*Mmm*) have been described yet.

*Mmm* is the causative agent of contagious bovine pleuropneumonia (CBPP), a severe, highly contagious respiratory disease of cattle. The disease has also been reported in Italian buffaloes (*Bubalus bubalis*) [8], American bison (*Bison bison*) and Asian yak (*Bos grunniens*), but never in African buffalo (*Syncerus caffer*) [9]. CBPP is transmitted via aerosols of infected animals and is characterized by severe inflammatory, exudative lesions at lung and pleural membranes. In calves however, infection of *Mmm* results mainly in swollen painful limbs (arthritis) and associated lameness, but rarely in pulmonary lesions [9]. *Mmm* belongs to the closely related *M. mycoides* cluster [10] which includes four other species and subspecies, namely *M. mycoides* subsp. *capri* (*Mmc*), *M. capricolum* subsp. *capripneumoniae*, *M. capricolum* subsp. *capricolum*, and *M. leachii*. Although *Mmc* and *Mmm* are phylogenetically closely related [11], they greatly differ in host tropism [12]. Unlike *Mmm*, *Mmc* affects small ruminants causing clinical signs including mastitis, arthritis, kerato-conjonctivitis, pneumonia and septicemia, abbreviated as ‘MAKePS’ [13]. *Mmm* has been isolated from sheep and goats [14] however there is no indication that it can cause disease in these species. Likewise, *Mmc* has also been isolated from cattle [15], but has not been reported to cause disease in cattle.

A previous study [16] demonstrated the adherence of *Mmm* to embryonic calf nasal epithelial (ECaNEp) cells using realtime PCR assays. Flow cytometric assays have been previously described to measure cyto-adherence of other *Mycoplasma* species [17,18]. The aim of this study was to set up a novel flow cytometry (FCM)-based high throughput adhesion screening for *Mmm* and utilize this model for characterization of adhesion among different ruminant cell lines and a panel of *M. mycoides* strains. This approach might reveal an *in vitro* binding pattern that correlates with disease outcomes observed in young and adult cattle.

## Methods

### Cell culture

Primary epithelial and endothelial cells were cultured from adult bovine tissues including accessory lung lobe epithelial cells (BoLEC) and aorta endothelial cells (BoAEC), bovine fetal lung epithelial cells (BoFLEC) and caprine lung epithelial cells (CaLEC) were cultured from bovine fetuses and goat lungs respectively. Additionally, previously established immortal bovine skin fibroblasts (IBoSF) were used in this study [19].

BoLEC and CaLEC were cultured using the protease digestion technique as described elsewhere [20]. Briefly, tissues were digested using 1% protease XIV (Sigma, St Louis, USA) at 4°C overnight before collecting cells. BoAEC were cultured using the same technique as described above however tissues were digested in 0.25% collagenase (Sigma, St Louis, USA) [21]. BoFLEC were cultured according to a method described by Schweizer and Peterhans (1999) [22] with slight modifications. Fetal lung tissue was digested in trypsin-EDTA (0.025%: 0.01%) in PBS at 37°C for 1 h with shaking (200 rpm). All cell lines were maintained in Dulbecco’s minimum essential medium (DMEM) supplemented with 10% inactivated fetal bovine serum (Sigma, St Louis, USA), 200 IU/ml penicillin, 150 μg/ml streptomycin, 1 μ g/ml nystatin, 2 mM L-glutamine and 0.15 M 2-mercaptoethanol.

### *Mycoplasma* culture preparations

*Mmm* and *Mmc* strains used in this study (Table 1) were cultured in 20 ml “pleuropneumonia -like organism” (PPLO) medium (Becton Dickinson, Park, USA) supplemented with 10% horse serum (Sigma, St Louis, USA), 0.9 g/l yeast extract, 0.5% glucose and 0.03% penicillin G at 37°C for 48 and 18 h to a density of 10^8^ and 10^9^ cfu/ml respectively. Each *Mycoplasma* strain concentration was determined by the Spearman–Karber formula [23] before centrifugation. The mycoplasmas were harvested by centrifugation at 6,000 × g at 4°C for 30 min, washed once in DMEM without supplements and suspended in 10 ml of the same media. Final *Mycoplasma* titers were calculated using optical density at OD650 and readings plotted on a standard curve based on correspondence between OD650 and *Mmm* numbers as determined by TaqMan Real Time PCR.

**Table 1:** ***Mycoplasma*** strains used in this study*

**Species**		**Strain name**	Other name**	**Date of isolation**	**Country of isolation**	**Host**	**Provider** ^**+**^
*Mycoplasma mycoides* subsp *mycoides* (*Mmm*)		Afade	DL 06/06	1968	Cameroon	Cattle/lung	FLI
		B237	DL 04/09	1997	Kenya		KARI
		B66		2000		Cattle	
		T144	DL 12/06	1951	Tanzania	Cattle/vaccine strain	CIRAD
		V5	DL 641/08	1936	Australia		FLI
		Gladysdale	008/06	1953		Cattle	
		Madrid	636/08	1993	Spain		
		L2	008/07	1993	Italy	Cattle/lung	
*Mycoplasma mycoides* subsp *capri* (*Mmc*)	*Mmc* serovar	PG3	R 88	1950	Turkey	Goat/type strain	
		Capri-L	402/97	1975	France	Goat	
	*Mmm* LC serovar	83/93	387/94	1993	Spain		
		136/93	383/94	1993			
		171/93	388/94	1993			
		152/93	385/94	1993			
		My325	64/97	1986	Croatia		
		G1313.94	211/94	1994	Germany	Sheep	
		G1255.94	209/94	1994			

*Strain selection was done to include a wide geographical location, varied years of isolation and different virulence.

^+^FLI-Friedrich Loeffler Institute; KARI- Kenya Agricultural Research Institute; CIRAD- Centre de coopération internationale en recherche agronomique pour le développement.

**All other names are FLI names.

### Polyclonal rabbit antibody production

The production of rabbit polyclonal antibodies was approved by the institutional animal care and use committee (IACUC reference number 2008.14). *Mmm* strain Afadé was cultured as described above and the proteins prepared by ultra-sonication, and protein concentration determined by the micro BCA according to the manufacturer’s instructions. One six weeks old rabbit was immunized twice with 1 mg of whole cell lysate at an interval of eight weeks. The rabbit was immunized by giving two immunizations with heat inactivated (60°C for 10 min) whole cell lysate (500 μl) mixed with the same volume of complete Freund’s adjuvant in the first immunization or the same volume of incomplete Freund’s adjuvant in the boost. At each immunization half of the antigen-adjuvant mixture was administered intradermally (500 μl) and half subcutaneously (500 μl). Pre-immunization serum was collected two weeks before initial immunization. Post-immunization serum was collected four weeks after the second immunization, heat inactivated at 56°C for 30 min, aliquoted and stored at -20°C until use.

The rabbit serum was validated using dot blot for all the *Mycoplasma* strains used in this study. Briefly, 2 μl of proteins from each *Mycoplasma* strain was dotted on to nitrocellulose membrane (0.45 μm pore size, Bio-rad), and allowed to dry at room temperature for 30 min. Unspecific binding sites on the blot were blocked in 5% BSA-PBS for 1 hr at room temperature. The blot was then incubated for 1 hr at room temperature with the rabbit serum diluted at 1:2,500. After 3 washes in PBS-Tween 20, the blots were incubated for 1 hr at room temperature with anti-rabbit IgG (whole molecule)-alkaline phosphatase antibody produced in goat (Sigma) at a dilution of 1:5000. After three washes, blots were developed with 5-bromo-4-chloro-3-indolyl phosphate/nitro blue tetrazolium (BCIP/NBT, Sigma) in alkaline phosphatase buffer (100 mM NaCl, 5 mM MgCl_2_, 100 mM Tris, pH 9.5).

### Indirect immunofluorescent microscopy

1×10^5^ BoLEC resuspended in 500 μl complete DMEM were seeded into a six cell well plate (Corning, New York, USA) containing sterile glass cover slips (Thermo Scientific) and cultured for 2-3 days at 37°C in 5% CO_2_. Cover slips were collected and washed three times in DMEM without supplements. *Mmm* strain Afadé was cultured as described above and 200 μl (approximately 1.5×10^8^ mycoplasmas) was added to the cell culture and incubated for two hours. Unbound mycoplasmas were washed off three times using 500 μl DMEM without supplements, prewarmed to 37°C. Cells were fixed in 3% paraformaldehyde for 15 min at room temperature. Cyto-adherent mycoplasmas were indirectly stained using anti-*Mmm* Afadé polyclonal rabbit serum (100 μl, 1:100 dilution) for 1 hour on ice, followed by washing thrice with 500 μl cold DMEM without supplements and 1 hour incubation with goat anti-rabbit FITC (Sigma, St Louis, USA; 100 μl, 1:500 dilution). Cover slips were air dried and images acquired using a fluorescent microscope (Axio Imager, Carl Zeiss AG, Gottingen, Germany) at a magnification of 400x. A control experiment was done as described above only that mycoplasmas were omitted and the cells were counter stained with 4′, 6 –Diamidino-2-phenylindole (DAPI).

### Flow cytometry analysis of cyto-adherence

Cell lines were cultured in 24 well plates to a density of approximately 1.5×10^5^ cells per well. To determine the optimum minimum concentration of mycoplasmas for cyto-adherence, all the *Mycoplasma* strains used in this study were ten-fold serially diluted. The cyto-adherence assay was performed as described above. Unbound mycoplasmas were washed off thrice with 500 μl prewarmed DMEM without supplements. Cells with bound mycoplasmas were detached from the culture plates with 250 μl PBS/EDTA buffer and the reaction stopped after 3 min by adding 500 μl prewarmed DMEM without supplements, transferred to 1.5 ml eppendorf tubes and centrifuged at 1,000 g for 10 min. The supernatant was discarded and the cells transferred to 96 round bottom well plates (Corning, New York, USA). Experiments were performed three times in duplicate. Staining was done as described above (100 μl/well, 1:100 dilution), followed by washing thrice with 200 μl of cold DMEM without supplements and centrifugation for 2 minutes at 1000 g. Cells were incubated with goat anti- rabbit FITC (Sigma, St Louis, USA; 100 μl/well, 1:250 dilution) for 1 hour on ice. After the final wash, cells were suspended in 100 μl of FACS medium (PBS, 2% horse serum, 0.2% sodium azide and 2% formalin). Cells were analysed by flow cytometry (FACS canto II, Becton Dickinson Co., San Jose, USA) in 5 ml falcon tubes according to the manufacturer’s instructions. Three thousand signals were acquired and the results presented as dot plots. To calculate the percentage of positive cells, fluorescence threshold was set such that unstained cells without mycoplasmas would have 0% fluorescence. This threshold was kept the same for all samples of each experiment and reset for each cell type. Percentage of cells with bound mycoplasmas and mean fluorescence intensity (MFI) were calculated using flow jo software [24]. Statistical analysis of the cells with bound mycoplasmas was done using ANOVA.

## Results

### Specificity and kinetics of cyto-adherence

Rabbit serum directed against whole cell antigens of *Mmm* reacted to both *Mmm* and *Mmc* with similar efficiency (see Additional file 1: Figure S1), which justified its subsequent use for the detection of both *Mycoplasma* subspecies.

**Figure 1:**
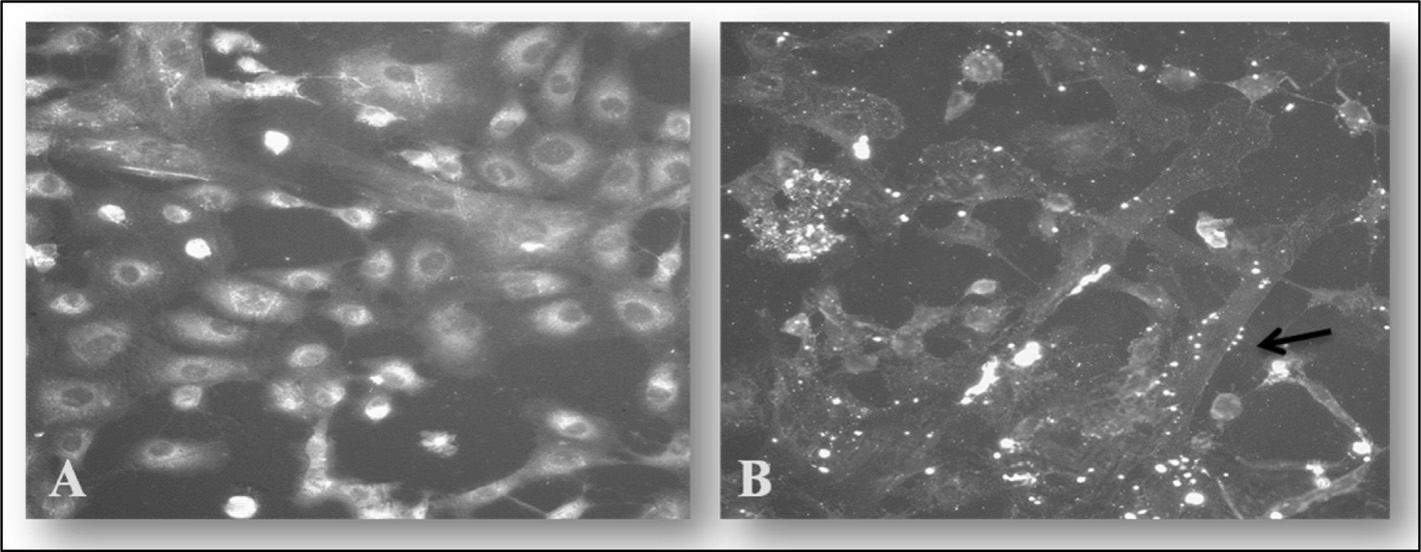
**Indirect immunofluorescent staining of** ***Mycoplasma mycoides*** **subsp**. ***mycoides*** **(** ***Mmm*** **) bound to bovine lung epithelial cells (BoLEC).** **A** shows BoLEC without *Mmm* stained with anti-*Mmm* (Afade) + anti-rabbit FITC and counter stained with DAPI. **B** shows BoLEC with adherent *Mmm* stained with anti-*Mmm* (Afade) + anti-rabbit FITC and counter stained without DAPI. The arrow indicates bright fluorescent patches of *Mycoplasma* clusters on the surface of an epithelial cell.

Microscopic analysis of adherent *Mmm* to BoLEC showed uneven distribution of *Mmm* on the cell surface. The adherent mycoplasmas appeared as aggregated bright fluorescent spots on the surface of the cells (Figure 1). Due to the small size of *Mmm*, it was not possible to visualize individual *Mycoplasma* cells under the conditions used.

**Figure 2:**
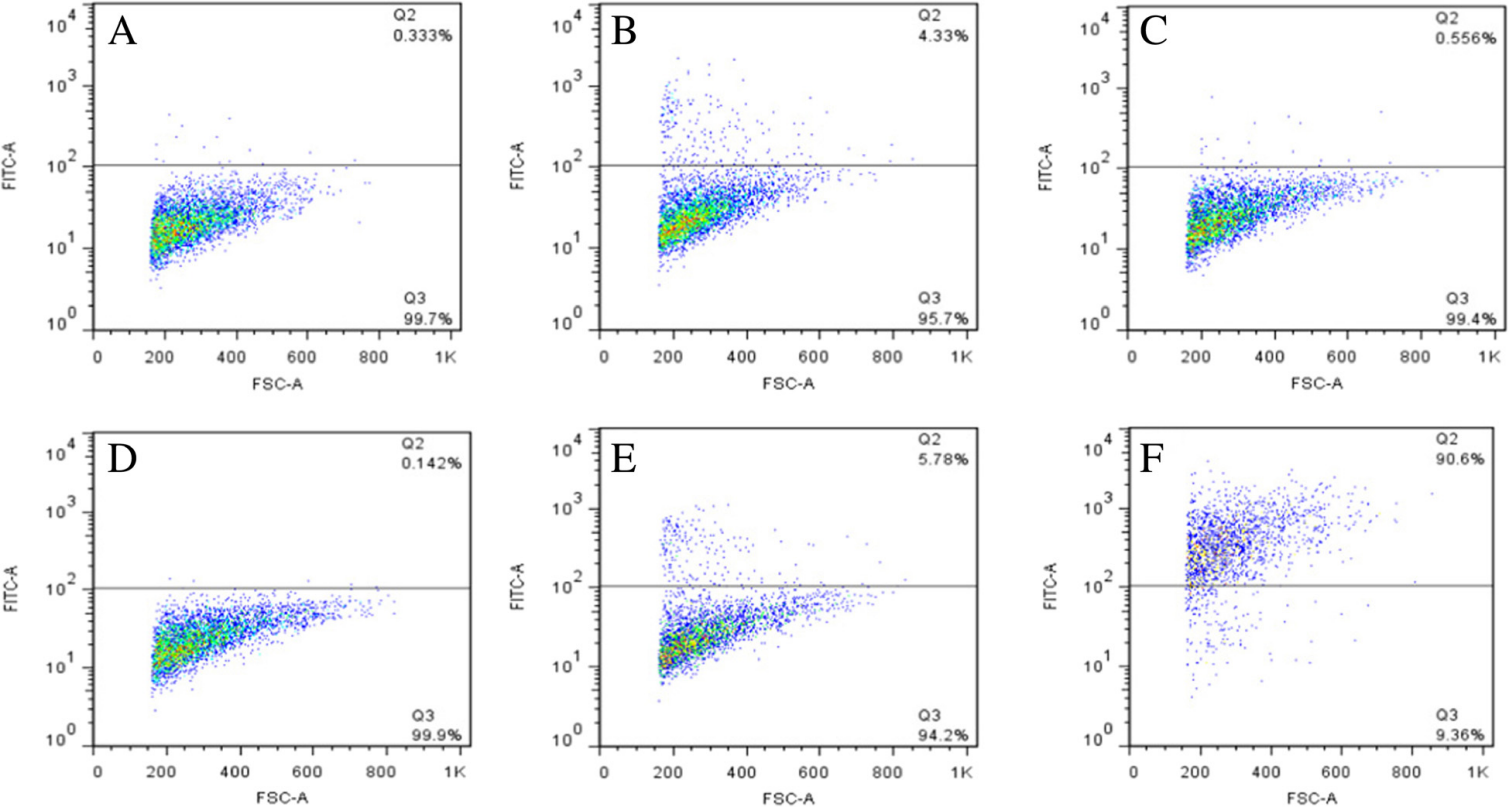
**Flow cytometric analysis of** ***Mycoplasma mycoides*** **subsp.** ***mycoides*** **(** ***Mmm*** **strain Afade) attachment to bovine lung epithelial cells (BoLEC).** Adherent *Mmm* was detected by specific rabbit anti-*Mmm* serum followed by FITC-conjugated goat anti-rabbit-Ig **(A)** unstained BoLEC without *Mmm*; **(B)** stained BoLEC without *Mmm*; **(C)** BoLEC + *Mmm* without anti-*Mmm* rabbit serum; **(D)** BoLEC + *Mmm* + anti-*Mmm* rabbit serum without goat anti- rabbit FITC; **(E)** BoLEC + *Mmm* + pre-immunization rabbit serum + goat anti-rabbit FITC; **(F)** BoLEC + *Mmm* + anti-*Mmm* rabbit serum + goat anti-rabbit FITC. Vertical axis: relative fluorescence intensity. Horizontal axis: forward light scatter.

**Figure 3:**
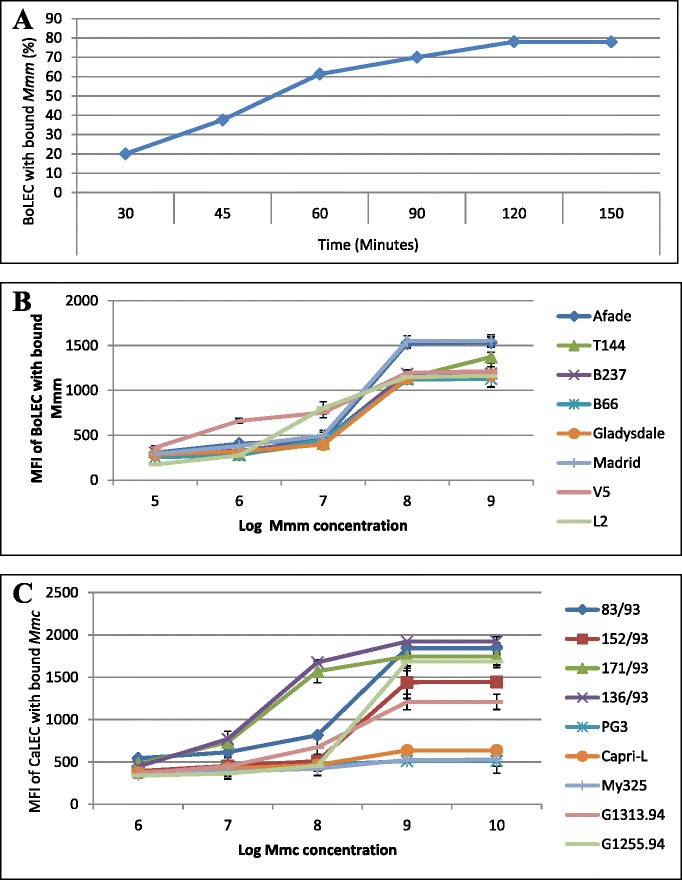
**Cyto**-**adhesion kinetics of** ***Mycoplasma*** **strains used in this study. (A)** Adherence of *Mycoplasma mycoides* subsp. *mycoides* (*Mmm*) strain Afade to cattle lung epithelial cells (BoLEC) as a function of incubation time. **(B)** Serial dilution (1:10) of *Mmm* strains and adherence to BoLEC. **(C)** Serial dilution (1:10) of *Mycoplasma mycoides* subsp. *capri* (*Mmc*) strains and adherence to caprine lung epithelial cells (CaLEC).

*Mmm* cyto-adherence kinetics were analyzed using an indirect flow cytometry assay with appropriate controls using unstained lung epithelial cells without adherent mycoplasmas to set the fluorescence cut-off level (Figure 2). In all subsequent experiments the MFI was used to compare binding of *Mmm* to target cells, as this was a more representative parameter of the relative average number of mycoplasmas bound per cell. Saturation of *Mmm* binding to BoLEC and *Mmc* to CaLEC occurred 2 hours post infection (Figure 3A) at approximately 10^8^ and 10^9^ mycoplasmas per 1.5×10^5^ cells (Figure 3B and C). In all subsequent experiments, mycoplasmas from each strain were used at saturating numbers.

**Figure 4:**
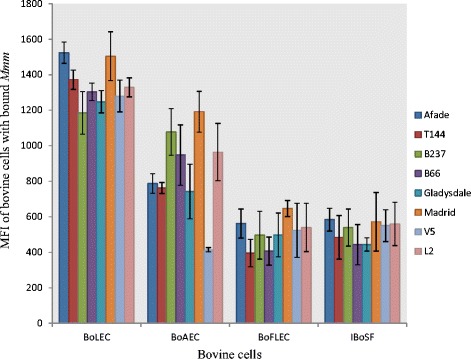
**Cyto-** **adherence capacity of different** ***Mycoplasma mycoides*** **subsp.** ***mycoides*** **(** ***Mmm*** **) strains to different cattle cell lines, expressed as mean fluorescence intensity (MFI) of cells with bound** ***Mycoplasma*** **.**

Cell lines from different host tissues were cultured to assess the binding specificity of a panel of eight *Mmm* strains (Table 1). Maximum binding of all *Mmm* strains occurred on BoLEC, whereas intermediate binding occurred with BoAEC (p = 0.006) and low binding with BoFLEC (p = 2.12 × 10^-10^) and IBoSF (p = 7.24 × 10^-11^) (Figure 4).

**Figure 5:**
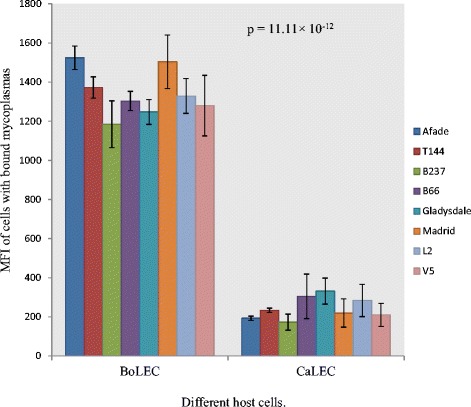
**Cyto-** **adherence capacity of different** ***Mycoplasma mycoides*** **subsp.** ***mycoides*** **(** ***Mmm*** **) strains to lung epithelial cells from cattle (BoLEC) and goats (CaLEC), expressed as mean fluorescence intensity (MFI) of cells with bound** ***Mycoplasma*** **.**

**Figure 6:**
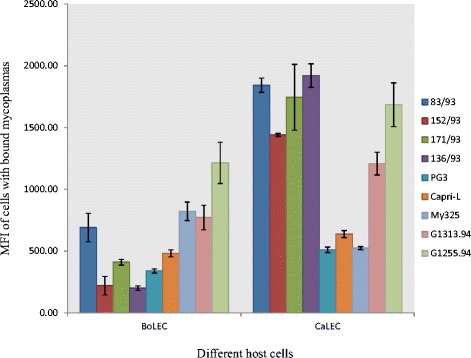
**Cyto-** **adherence of different** ***Mycoplasma mycoides*** **subsp.** ***capri*** **(** ***Mmc*** **) strains to lung epithelial cells from cattle (BoLEC) and goats (CaLEC), expressed as mean fluorescence intensity (MFI) of cells with bound** ***Mycoplasma*** **.**

Cyto-adherence of *Mmm* and *Mmc* to specific host cells was assessed by testing the binding ability of both *Mycoplasma* species to goat and cattle cells. We demonstrated a statistically significant difference between the cyto-adherence capacities of *Mmm* to BoLEC and CaLEC (p = 1.11 × 10^-12^), with *Mmm* binding more efficiently to BoLEC than CaLEC (Figure 5). On the other hand, *Mmc* bound more efficiently to CaLEC than BoLEC (Figure 6) however the difference was less apparent than in the case of *Mmm* but statistically significant (p = 0.006).

### Intraspecies variation of cyto-adherence capacity

The different strains of *Mmm* did not show much variation in the ability to adhere to BoLEC although some small statistical differences of cyto-adherence capacities could be detected for certain strains; in particular with strain Madrid which seemed to have a higher binding capacity compared to the other strains (Figure 5; see Additional file 2: Table S1). *Mmc* on the other hand showed relatively high intra-species variation in its capacity to adhere to CaLEC with strains PG3, Capri-L and My325 binding weakly to the cells (Figure 6; see Additional file 3: Table S2).

## Discussion

The aim of this study was to set up a novel flow cytometry (FCM)-based high throughput screening for adhesion of *Mmm* and to utilize the model for characterization of adhesion of various strains of *M. mycoides* subsp. *mycoides* and *M. mycoides* subsp. *capri* to different ruminant cell lines. In our study, specks of fluorescence could be detected that varied in size, suggesting that *Mmm* were often present as clusters or aggregates of different sizes. A previous study also showed a heterogeneous distribution of *M. bovis* on the surface of host cells [20]. Using the flow cytometric assay we determined a statistical difference in the binding of *Mmm* to various bovine host tissues; the highest binding was detected with BoLEC, intermediate binding with BoAEC and low binding with IBoSF and BoFLEC. High binding to BoLEC is expected as the main lesions and pathology caused by *Mmm* are found in the lung of adult cattle [25] suggesting that there might be a receptor for *Mmm* on lung cells that allows for this interaction. Low adherence of *Mmm* to fetal lung epithelial cells may suggest a lack or low abundance of a receptor for *Mmm*, which may be acquired through activation of the corresponding genes later in life. This result probably explains why neonatal calves that are infected with *Mmm* do not develop severe lung lesions [9]. This also correlates with epidemiological observations showing that CBPP generally affects adult cattle [9,12]. In a previous study, binding of *Mmm* to embryonic calf nasal epithelial cells (ECaNEp) was measured in order to analyze the differences in the adhesion capacity of *Mmm* strains with diverse degrees of virulence, as ECaNEp cells were used as a model for cytotoxicity. However, this study did not compare host cells of animals of various ages and hence measurements have been done at a relatively low range of binding capacity [16].

Moderate binding to endothelial cells might correlate with pathology seen in capillaries and lymph vessels. *Mmm* has been isolated from the lymph nodes of cattle with CBPP [25] and vasculitis is a common occurrence in heavily infected animals. An interaction between mycoplasmas and endothelial cells may contribute to the vasculitis. Subcutaneous inoculation of mycoplasma often causes serious necrotic edema, but not in lymphatic-poor areas, such as nose or tail tip [26] however this method of inoculation does not result in typical clinical CBPP.

According to our results, there was little significant statistical difference between the adherence capacities of the different *Mmm* strains tested, which suggests that all strains used expressed the relevant adhesion ligands and the differences in the virulence among these strains is not due to variation in their adhesins. This is also probably due to the fact the *Mmm* strains exhibit very little genetic diversity [11]. The caprine *Mycoplasma* (*Mmc*) on the other hand showed more variation in cyto-adherence patterns among strains, which could correlate with their broader genetic diversity [11]. But the clinical signs of *Mmc* are not confined to lung, as there are many other manifestations, abbreviated as ‘MAKePS’ [13]. Adherence to other cell types might show different patterns and assessing the correlations between cyto-adherence of *Mmc* strains and different cell types needs further investigation. Furthermore, there is a possibility that the rabbit anti-Mmm serum used in this study might have reacted differently with individual *Mmc* strains. *Mmm* cyto-adherence assays revealed relevant host species specificity: *Mmm* adheres to bovine lung epithelial cells, but very little to caprine lung epithelial cells. This suggests a correlation between the capacity to adhere to target cells, in this case to cattle cells, and the development of disease specifically in cattle, suggesting the presence of host specific adhesion factors. However, *Mmc* binds to lung cells from both species. This needs further investigation since *Mmc* has never been reported as causing disease in cattle [15] but other factors may influence the disease outcome. The cyto-adherence of *Mmm* supports previous reports from other *Mycoplasma* species that adherence is tissue and organ specific; however it is not uncommon to isolate mycoplasmas from tissues or hosts other than their natural habitats [27].

## Conclusion

Cyto-adherence of *Mmm* to mammalian host cells was studied using an indirect, flow cytometric assay. The data show that strong binding of *Mmm* is specific to lung epithelial cells from adult cattle, but not fetuses. This correlates with the severe *in vivo* clinical signs observed in lung tissue of infected, adult cattle, but which are mostly absent in infected, very young calves. An intermediate binding to endothelial cells correlates with pathological signs detected in capillaries and lymph vessels. Binding was weak to other tissue cells and to lung cells from goats, suggesting that a strong binding of *Mmm* with their target cells might be the basis of the species and tissue specificity of CBPP, and be a requisite step in the development of the disease. Blocking adhesion *in vivo* might represent a valuable target to prevent colonization of the lung by *Mmm*.

## Additional files

Additional file 1: Figure S1. Dot blot analysis of the ability of rabbit serum to recognize all the strains used in this study. 1. Blank control. No. 2-9. *Mycoplasma mycoides* subsp. *mycoides* strains including Afade, T144, B237, B66, Gladysdale, Madrid, V5 and L2 respectively. Numbers 10-18 are *Mycoplasma mycoides* subsp. *capri* strains including 83/83, 152/93, 171/93, 136/93, PG3, Capri-L, My325, G1313.94 and G1255.94 respectively. Membrane stained with *Mmm* specific rabbit serum raised against strain Afade (1:2500) and goat anti-rabbit alkaline phosphatase conjugated (1:500) (Sigma) and visualized by 5-bromo-4-chloro-3-indolyl phosphate/nitro blue tetrazolium (BCIP/NBT, Sigma). Additional file 2: Table S1. Statistical analysis of cyto-adherence capacity of *Mycoplasma mycoides* subsp. *mycoides* (*Mmm*) strains to bovine lung epithelial cells (BoLEC). Additional file 3: Table S2. Statistical analysis of *Mycoplasma mycoides* subsp. *capri* (*Mmc*) strains cyto-adherence to goat lung epithelial cells (CaLEC).

## Abbreviations

BoAEC: Bovine aortic endothelial cells
BoFLEC: Bovine fetal lung epithelial cells
BoLEC: Bovine (adult) lung epithelial cells
CaLEC: Caprine lung epithelial cells
CBPP: Contagious bovine pleuropneumonia
IBoSF: Immortalized (bovine) skin fibroblast
Mmm: *Mycoplasma mycoides* subsp. *mycoides*
*Mmc*: *Mycoplasma mycoides* subsp. *capri*
DMEM: Dulbecco’s minimum essential medium
DAPI: 4′, 6-Diamidino-2- phenylindole
ECaNEp: Embryonic calf nasal epithelial cells
