# Supplementary figures and images for: Cyto-adherence of Mycoplasma mycoides subsp. mycoides to bovine lung epithelial cells
Source: BMC Vet Res. 2015 Feb 7;11:27. doi: 10.1186/s12917-015-0347-3 (PMC4336739; doi:10.1186/s12917-015-0347-3)

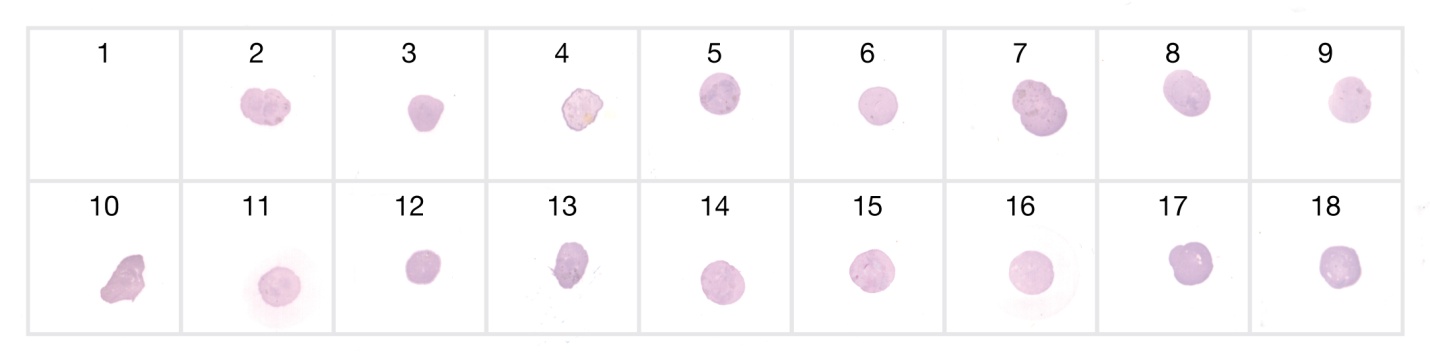


Additional figure 1

Supplement: Additional file 1: Figure S1. — Dot blot analysis of the ability of rabbit serum to recognize all the strains used in this study. 1. Blank control. No. 2-9. Mycoplasma mycoides subsp. mycoides strains including Afade, T144, B237, B66, Gladysdale, Madrid, V5 and L2 respectively. Numbers 10-18 are Mycoplasma mycoides subsp. capri strains including 83/83, 152/93, 171/93, 136/93, PG3, Capri-L, My325, G1313.94 and G1255.94 respectively. Membrane stained with Mmm specific rabbit serum raised against strain Afade (1:2500) and goat anti-rabbit alkaline phosphatase conjugated (1:500) (Sigma) and visualized by 5-bromo-4-chloro-3-indolyl phosphate/nitro blue tetrazolium (BCIP/NBT, Sigma). [file 12917_2015_347_MOESM1_ESM.docx]
