# Supplementary material for: Cyto-adherence of Mycoplasma mycoides subsp. mycoides to bovine lung epithelial cells
Source: BMC Vet Res. 2015 Feb 7;11:27. doi: 10.1186/s12917-015-0347-3 (PMC4336739; doi:10.1186/s12917-015-0347-3)
Supplement: Additional file 2: Table S1. — Statistical analysis of cyto-adherence capacity of Mycoplasma mycoides subsp. mycoides (Mmm) strains to bovine lung epithelial cells (BoLEC). [file 12917_2015_347_MOESM2_ESM.docx]

**Additional table 1:** Statistical analysis of cyto-adherence capacity of *Mycoplasma mycoides* subsp *mycoides* (*Mmm*) strains to bovine lung epithelial cells (BoLEC)

|  | **Afade** | **T144** | **B237** | **B66** | **Gladysdale** | **Madrid** | **V5** |
| --- | --- | --- | --- | --- | --- | --- | --- |
| **Afade** |  |  |  |  |  |  |  |
| **T144** | 0.59 |  |  |  |  |  |  |
| **B237** | 0.83 | 0.053 |  |  |  |  |  |
| **B66** | 0.91 | 0.11 | 0.123 |  |  |  |  |
| **Gladysdale** | 0.88 | 0.062 | 0.39 | 0.22 |  |  |  |
| **Madrid** | 0.38 | 0.13 | 0.014* | 0.04* | 0.016* |  |  |
| **V5** | 0.078 | 0.45 | 0.108 | 0.64 | 0.2 | 0.08 |  |
| **L2** | 0.074 | 0.056 | 0.21 | 0.55 | 0.47 | 0.02* | 0.39 |

*Significant statistical difference in the cyto-adherence capacity of the *Mmm* strains to BoLEC
