# Supplementary material for: Cyto-adherence of Mycoplasma mycoides subsp. mycoides to bovine lung epithelial cells
Source: BMC Vet Res. 2015 Feb 7;11:27. doi: 10.1186/s12917-015-0347-3 (PMC4336739; doi:10.1186/s12917-015-0347-3)
Supplement: Additional file 3: Table S2. — Statistical analysis of Mycoplasma mycoides subsp. capri (Mmc) strains cyto-adherence to goat lung epithelial cells (CaLEC). [file 12917_2015_347_MOESM3_ESM.docx]

**Additional table 2:** Statistical analysis of *Mycoplasma mycoides* subsp *capri* (*Mmc*) cyto-adherence to goat lung epithelial cells (CaLEC)

|  | **83/93** | **152/93** | **171/93** | **136/93** | **PG3** | **Capri-L** | **My325** | **G1313.94** |
| --- | --- | --- | --- | --- | --- | --- | --- | --- |
| **83/93** |  |  |  |  |  |  |  |  |
| **152/93** | 0.0002 |  |  |  |  |  |  |  |
| **171/93** | 0.17* | 3.7×10^-5^ |  |  |  |  |  |  |
| **136/93** | 0.0026 | 0.04 | 0.0004 |  |  |  |  |  |
| **PG3** | 6.78×10^-6^ | 2.72×10^-7^ | 3.67×10^-6^ | 1.14×10^-6^ |  |  |  |  |
| **Capri-L** | 2.58×10^-6^ | 1.03×10^-7^ | 7.63×10^-7^ | 5.05×10^-7^ | 0.41* |  |  |  |
| **My325** | 1.04×10^-5^ | 2.32×10^-7^ | 3.78×10^-6^ | 1.3×10^-6^ | 0.0014 | 2.45×10^-2^ |  |  |
| **G1313.94** | 0.21* | 0.007 | 0.05 | 0.08 | 4.63×10^-5^ | 3.07×10^-5^ | 3.08×10^-5^ |  |
| **G1255.94** | 0.73* | 3.28×10^-5^ | 0.022 | 0.0007 | 1.03×10^-7^ | 3.1×10^-11^ | 3.11×10^-11^ | 0.21* |

*No significant statistical difference between the cyto-adherence of the *Mmc* strains to CaLEC
